# Children with Attention Deficit/Hyperactivity Disorder and Reading Disability: A Review of the Efficacy of Medication Treatments

**DOI:** 10.3389/fpsyg.2016.00988

**Published:** 2016-07-05

**Authors:** Christina Gray, Emma A. Climie

**Affiliations:** Werklund School of Education, University of CalgaryCalgary, AB, Canada

**Keywords:** attention-deficit/hyperactivity disorder, ADHD, reading disability, SLD-R, intervention, medication treatment, methylphenidate, atomoxetine

## Abstract

Reading is a multifaceted skillset that has the potential to profoundly impact a child’s academic performance and achievement. Mastery of reading skills is often an area of difficulty for children during their academic journey, particularly for children with Attention Deficit/Hyperactivity Disorder (ADHD), Specific Learning Disorder with Impairment in Reading (SLD-R), or children with a comorbid diagnosis of both ADHD and SLD-R. ADHD is characterized by executive functioning and impulse control deficits, as well as inattention and impulsivity. Among the academic struggles experienced by children with ADHD are challenges with word reading, decoding, or reading comprehension. Similarly, children with SLD-R frequently encounter difficulties in the development of appropriate reading skills. SLD-R incorporates dysfunctions in basic visual and auditory processes that result in difficulties with decoding and spelling words. There have been limited empirical studies investigating the efficacy of interventions to improve the reading ability of children with both ADHD and SLD-R. Research studies that have focused on reading interventions for children from this population have predominantly included the use of medication treatments with stimulants (e.g., methylphenidate) and non-stimulants (e.g., atomoxetine). This review paper will present and integrate findings from empirical studies on successful medication treatments for children with comorbid ADHD and SLD-R. Furthermore, this paper will extend findings from empirically successful medication treatments to provide directions for future research.

## Reading

Developing reading competency is a critical learning milestone for children. Reading is a multifaceted skillset that has potential to profoundly impact a child’s academic achievement. It is a complex process consisting of two primary components: word recognition (decoding words and sight word reading) and comprehension (understanding; Aaron et al., 1999). In learning to read, children move through the process of letter-sound identification, building phonemes, and enhancing their phonetic understanding (Lewandowski and Lovett, 2014). Once children have mastered these pre-reading skills, they are able to identify and decode individual words. Building on these skills, reading comprehension involves understanding the meaning of decoded words (Aaron et al., 2002), with the goal of developing reading fluency. Word recognition and comprehension develop independently and difficulties with reading can result from either underdeveloped decoding skills or weak comprehension skills (Aaron et al., 2002).

The process of reading is additionally dependent on the reader’s language comprehension, expressive language skills, and overall metalinguistic ability (i.e., the ability to conceptualize language as an object of thought). Thus, attaining a level of success as a reader requires proficiency in a range of foundational and interrelated skills whereby the reader uses knowledge of syntax, semantics, pragmatics, and phonology (Van Kleeck and Schuele, 1987). Given the interconnection between language and learning, reading mastery is essential for academic success. Children who lack early reading and literacy skills are at greater risk of experiencing later difficulties with reading than children who make developmentally appropriate gains (Scanlon and Vellutino, 1996). Successful mastery of reading skills is frequently an area of difficulty for some children, sometimes resulting in a child being identified as having a reading disability or SLD-R.

Specific learning disorder (SLD) collapses the previously used terms of “learning disorders” (LD) and “academic skills disorders”, and includes impairments in the domains of reading, written expression, and mathematics (American Psychiatric Association [APA], 2013). SLDs are domain specific, with a majority of SLD identifications occurring in the area of reading. A child with significant academic impairments in the areas of word reading accuracy, reading rate/fluency, and/or reading comprehension may meet diagnostic criteria for SLD-R (American Psychiatric Association [APA], 2013). When children begin the process of learning how to read, they are expected to increase in accuracy and fluency with identification of the sound-symbol associations of words; however, children with SLD-R often struggle with this process of word decoding (Sheikhi et al., 2013). As a complex neurobehavioral disorder, SLD-R consists of dysfunction in basic visual and auditory processes that may impede language phonology and result in difficulties with decoding, interpreting, and spelling words (Sheikhi et al., 2013; Lewandowski and Lovett, 2014). Given the heterogeneous nature of SLDs, children with these challenges are frequently diagnosed with other disorders, such as ADHD (Barkley, 2006; Kaplan et al., 2006; American Psychiatric Association [APA], 2013).

## Attention Deficit/Hyperactivity Disorder

ADHD is a neurodevelopmental disorder that is characterized by executive functioning (EF) and impulse control deficits, as well as inattention and impulsivity (Barkley, 2014; Nigg and Barkley, 2014). Children with ADHD experience developmentally inappropriate levels of inattention and impulsivity that contribute to difficulties with daily functioning (Barkley, 2014). These children are often unable to sit still, struggle with staying on task, and talk at inappropriate times (Nigg and Barkley, 2014). These behaviors are particularly challenging in a classroom environment, where a lack of focus and attention may result in poor academic outcomes (Loe and Feldman, 2007).

### Executive Functioning and ADHD

In Barkley’s (1997) neuropsychological model of EF, behavioral inhibition allows for proficiency in four EF abilities: working memory (the ability to manipulate mental information), internalization of speech (the ability to “talk” oneself through a task), self-regulation of affect–motivation–arousal (the ability to manage ones’ emotions), and reconstitution/behavioral analysis and synthesis (the ability to reflect on ones actions). These EF abilities are subserved by the prefrontal cortex. Children with ADHD lack proficiency in carrying out these EF skills, demonstrated through difficulties with self-regulation skills such as planning ahead, problem solving, or self-monitoring.

It is estimated that ADHD affects between 8–20% of school aged children (Froehlich et al., 2007), while the prevalence of SLDs ranges between 5–15% (American Psychiatric Association [APA], 2013). The comorbidity rates of ADHD and SLD-R have been estimated to be between 31–45%, much greater than by chance (DuPaul et al., 2013; Stubenrauch et al., 2014). As a result of the substantial overlap between ADHD and SLD-R, it is critical to treat the disorders together rather than in isolation (Sexton et al., 2012).

### Reading Challenges in Children with ADHD

Reading is an effortful, complex task that requires sustained attention. Given the challenges associated with ADHD, particularly those related to EF, it is unsurprising that reading may be an area of difficulty for these children (Brock and Knapp, 1996). For example, children with ADHD often demonstrate difficulties in decoding, struggling with identifying written words (Willcutt et al., 2010; McGrath et al., 2011). EF skills are vital contributors to the process of developing reading skills. For example, working memory skills are required for decoding unfamiliar words, recalling previous text, and anticipating the storyline (Sesma et al., 2009). Planning and problem-solving skills are required when encountering unfamiliar words and critical analysis of passages, while organization is needed to understand the flow of a passage (Sesma et al., 2009). Processing speed is integral in the development of reading fluency (Mahone, 2011). Collectively, EF deficits, inattentiveness, and hyperactivity–impulsivity strongly impact a child with ADHD’s daily functioning, contributing to reduced academic attainment and school performance that are associated with the disorder (American Psychiatric Association [APA], 2013). This association suggests the presence of reading challenges for some children with ADHD may be due to neurological impairments that are either primarily (e.g., inattention) or secondarily (e.g., reading disorders) linked with ADHD.

Children with ADHD may also present with deficits in reading comprehension (Miller et al., 2013; Fienup et al., 2015). However, empirical studies examining reading comprehension abilities in children with ADHD produced mixed findings. Several studies have reported reading comprehension difficulties in children with ADHD (Cherkes-Julkowski et al., 1995; Brock and Knapp, 1996; Miller et al., 2013), while others have not (Ghelani et al., 2004). In those that noted difficulties, reading comprehension was found to decrease for children with ADHD when the length of a reading passage was increased, as compared to children with a reading disability or typically developing children (Cherkes-Julkowski et al., 1995). Children with ADHD also have lower reading comprehension scores and more difficulty reporting the central idea from a passage than those without ADHD. This difficulty may be due to the attentional demands of lengthy reading passages, requiring more effortful processing (Brock and Knapp, 1996). Additionally, children with ADHD show deficits with recalling central information in a text passage, most likely as a result of working memory difficulties (Miller et al., 2013). Conversely, Ghelani et al. (2004) found that adolescents with ADHD had sufficient single word reading abilities, and attained average scores on measures of text reading rate, accuracy, and silent reading comprehension. However, although the adolescents with ADHD attained scores in the average range, they were lower than those of normal controls. Collectively, the literature supports the presence of some difficulties in the reading skills of children with ADHD in a variety of areas, suggesting a need for remediation (Fienup et al., 2015).

## Treatment of ADHD/SLD-R

The following section provides a detailed review on what is known about treatments for children with comorbid ADHD and SLD-R (ADHD/SLD-R). Empirical studies and articles for this literature review were obtained through searches using the PsycINFO database, as well as Google Scholar to ensure inclusion of all relevant papers. The key search terms included *Attention-Deficit/Hyperactivity Disorder*, *reading disabilities*, *treatment*, and *medication*. The search of peer-reviewed articles was limited to years 1995–2016 to ensure that included papers were recent, given the advances in medication efficacy and treatment-based research in the past 20 years, and were included if they assessed the efficacy of medication treatments for children with comorbid ADHD/SLD-R. Studies that examined treatments for ADHD or SLD-R independently were not included. For comparison, a brief review of papers published prior to 1995 is included for comparison. Given the limited literature in the area of medication treatment for children with ADHD and SLD-R, only 6 relevant empirical papers have been included as the main focus of the review.

Much of the existing literature addressing treatments for children with ADHD have focused on remediating the core deficits associated with the disorder (e.g., inattention, impulsivity) rather than working to improve academic difficulties (Jitendra et al., 2008). However, empirical interventions designed to improve overall academic achievement for children with ADHD have included a focus on multimodal psychosocial treatment and consultation-based academic interventions (DuPaul and Stoner, 2003). For children with SLD-R, interventions frequently target decoding, fluency, and comprehension through strategy instruction (Manset-Williamson and Nelson, 2005). However, there has been limited investigation of the efficacy of treatments to improve reading outcomes in children with ADHD/SLD-R. In their review of literature on the co-occurrence of ADHD/RD, Sexton et al. (2012) suggest that pharmacotherapy (simulant or non-stimulant medication) in combination with behavioral or educational interventions should be used to best address the unique challenges these children face.

### Early Research On Medication And Reading Abilities

Historically, research in the 1980s and early 1990s yielded results questioning the efficacy medication treatments for children with reading disorders. Uncertainty existed over whether stimulant medication could also be used to aid academic performance rather than simply addressing behavioral challenges (Snider et al., 2003). A meta-analysis of stimulant medication studies from 1981 to 1995 found larger effect sizes for behavior outcomes, suggesting that stimulant medications did not significantly contribute to improvements in academic achievement (Forness et al., 1999). Several early studies found that stimulant medications were not associated with improvements in reading abilities (Forness et al., 1991, 1992). However, studies since this time have yielded results suggesting that medication treatments have the effect of improving reading outcomes in children with ADHD/SLD-R (Tannock et al., 2000; Keulers et al., 2007; Bental and Tirosh, 2008; Sumner et al., 2009; Shaywitz et al., 2014). The trending shift in findings relating to medication efficacy with treating ADHD/SLD-R suggests a need for further empirical study in this area.

### Stimulant Medication

Although stimulant medication is commonly used to treat the symptoms of ADHD (e.g., attention, focus; Barkley, 2014), its use in children with ADHD/SLD-R has only recently been considered. Stimulant medications, such as methylphenidate (trade names: Ritalin, Concerta), modulate EF thus improving cognitive attention and behavior (Evans et al., 2001; Grizenko et al., 2006). Findings from Grizenko et al. (2006) comparison of methylphenidate treatment response of children with ADHD to children with ADHD and broad LD found that 59% of children with ADHD and reading disability (ADHD/RD) displayed improvement with ADHD symptoms. These findings provided initial support for methylphenidate treatment for children with ADHD/RD, and additional studies have investigated the impacts of this treatment on improving reading performance in this population.

Given the enhancing effects of methylphenidate on EF, there is potential for stimulant medication to improve reading outcomes in children with ADHD/SLD-R (Tannock et al., 2000; Keulers et al., 2007; Bental and Tirosh, 2008). In particular, Tannock et al. (2000) examined the effects of simulant medication on a number of tasks known to be associated with reading, including color naming speed, letter naming speed, phonological decoding, and arithmetic computation. Although small effect sizes were found with methylphenidate selectively improving color-naming speed for children with ADHD and ADHD/RD, no impact was found with naming speed for alphanumeric stimuli, which is thought to play a vital role with word identification.

In addition, Keulers et al. (2007) also found that, following methylphenidate treatment, children with ADHD/dyslexia displayed increased reading outcomes, performing significantly better than those in control groups who did not receive treatment. Specifically, children with ADHD/dyslexia were able to correctly read a larger number of words, indicating that the reading process for children with ADHD/dyslexia may be aided with methylphenidate treatment.

Finally, methylphenidate has been found to contribute to improved word and non-word decoding accuracy as well as rapid naming (Bental and Tirosh, 2008). These researchers suggest that the improvements in decoding and naming in children with ADHD/RD may be due to the impact that methylphenidate has on enhancing cognitive attention. Collectively, these studies involving stimulant medication provide preliminary support for the use of methylphenidate as a possible treatment for children with ADHD/SLD-R, with demonstrated improvement in EF and attentional cognitive abilities. These improvements, in turn, may positively impact proficiency with aspects of reading.

### Non-stimulant Medication

The use of non-stimulant medications, particularly atomoxetine (trade name: Strattera), for improving reading skills in children with ADHD/SLD-R has also been examined. Atomoxetine has been found to effectively treat behavioral ADHD symptoms. Specifically, atomoxetine has been found to effectively reduce ADHD symptoms in children with ADHD only and ADHD/dyslexia (Sumner et al., 2009; Shaywitz et al., 2014). Improvements were found in decoding and spelling for both groups, and these improvements either were weakly correlated or did not correlate with the improvements found in ADHD-related symptoms (Sumner et al., 2009; Shaywitz et al., 2014).

Sumner et al. (2009) also found differences in patterns of reading and spelling score improvements following atomoxetine treatment in children with ADHD and ADHD/dyslexia. Improvements in spelling scores and greater working memory improvements were found for the ADHD-only group whereas the ADHD/dyslexia group displayed improvements in decoding. The significant difference found between groups suggests that the medication may have a differential neurological effect on the target areas in these groups of children.

Atomoxetine has also been found to improve visuospatial working memory and, to a lesser degree, inhibition in children with ADHD/RD (De Jong et al., 2009). It is thought that differential developmental pathways for ADHD/RD compared to ADHD or RD alone may explain improvements following atomoxetine treatments in this group. De Jong and colleagues found that atomoxetine treatment did not significantly affect reading performance itself, although ADHD symptoms were reduced. This differential effect of atomoxetine on reading performance and ADHD related symptoms may be due to neurocognitive factors contributing to covariance in both traits. In contrast, Shaywitz et al. (2014) found improvements in reading scores for children with ADHD/dyslexia following atomoxetine treatment. Specifically, Shaywitz and colleagues conducted correlation analyses which indicated that the improvements found in reading scores could not be fully explained by the decline in ADHD symptoms alone, suggesting non-stimulant medications may impact the reading performance of children with ADHD and reading difficulties.

Despite conflicting findings, results from recent studies involving stimulant and non-stimulant medication treatment options for children with ADHD/SLD-R optimistically suggest both forms of medication may contribute to improved reading outcomes. Although these medications do not directly target enhancement of reading skills, they play a role in supporting EF skills, such as working memory, processing speed, and attentional focus. These EF abilities play a key role in reading; thus, directly supporting these abilities at a neural level may allow children with ADHD/SLD-R to develop the building blocks needed to further their learning of reading-related skills.

### Directions For Research

Review of the literature on medication treatments to improve reading related challenges in children with ADHD/SLD-R has shown that stimulant and non-stimulant medications may be effective in treating ADHD symptoms (Vaughan et al., 2009). In particular, medication treatments have shown recent success in improving EF difficulties, which may, in turn, contribute to improvements in reading skills. However, the broad efficacy of medication treatments for children with ADHD/SLD-R has not received sufficient research attention (Sumner et al., 2009). When conducting research trials involving the effectiveness of medication treatments, it is ideal to utilize a blind, randomized-control, placebo trial. Although this methodological approach is not always feasible due to time or financial restraints, it may be worthwhile to replicate results from studies that did not use this research design to ensure accuracy of the findings. As well, the studies reviewed in this paper used differing methodologies, test subject composition, measures of ADHD symptoms, and measures of reading skills as dependent variables. Consistency in the reading and attention measures used may contribute to more conclusive findings and increased validity.

Another area sorely lacking research focus involves non-medicinal options for children with ADHD/SLD-R, with a need to build reading strategies or programs to improve literary skills in this population. Specifically, there is a need to consider the behavioral implications of ADHD in the creation of reading support programs for those with ADHD/SLD-R. For instance, inclusion of behavioral improvement strategies that focus on strengthening EF abilities in literacy programs may further benefit children with ADHD/SLD-R. Expansion of future research towards non-medicinal treatments may lead to effective alternative or concurrent treatment for children who do not respond to medication or for families who are looking for alternative options.

## Conclusion

Reading is a multifaceted process that combines mastery of word recognition and comprehension; proficient use of these skills is often a challenge for children with ADHD, SLD-R, and ADHD/SLD-R (Aaron et al., 2002; Willcutt et al., 2010). The importance of gaining adequate word reading and comprehension skills cannot be overlooked, given the importance of having basic reading and comprehension abilities. When an individual does not have adequate reading abilities, successful completion of high school and attainment of secure employment are significantly more challenging. It is necessary to work with those most at-risk of reading difficulties to ensure that they have the best opportunity to gain these necessary skills. Reading challenges are frequently a core difficulty for children with ADHD/SLD-R, and it is through evidence-based interventions that the academic functioning of these children can be best supported.

## Author Contributions

All authors listed, have made substantial, direct and intellectual contribution to the work, and approved it for publication.

## Conflict of Interest Statement

The authors declare that the research was conducted in the absence of any commercial or financial relationships that could be construed as a potential conflict of interest. The reviewers GKA and BS and handling Editor declared their shared affiliation; the handling Editor and Chief Editor both state that the process nevertheless met the standards of a fair and objective review.

## References

[B1] Aaron P. G. Joshi R. M. Palmer H. Smith N. Kirby E. (2002). Separating genuine cases of reading disability from reading deficits caused by predominantly inattentive ADHD behavior. *J. Learn. Disabil.* 35 425–436. 10.1177/00222194020350050301 15490539

[B2] Aaron P. G. Joshi R. M. Williams K. (1999). Not all reading disabilities are alike. *J. Learn. Disabil.* 32 120–137. 10.1177/002221949903200203 15499713

[B3] American Psychiatric Association [APA] (2013). *Diagnostic And Statistical Manual Of Mental Disorders*, 5th Edn. Washington, DC: American Psychiatric Publishing.

[B4] Barkley R. A. (2006). *Attention-Deficit Hyperactivity Disorder: A Handbook for Diagnosis and Treatment*, 3rd Edn New York, NY: The Guilford Press.

[B5] Barkley R. A. (1997). Behavioral inhibition, sustained attention, and executive functions: constructing a unifying theory of ADHD. *Psychol. Bull.* 121 65–94. 10.1037/0033-2909.121.1.65 9000892

[B6] Barkley R. A. (2014). *Attention-Deficit Hyperactivity Disorder: A Handbook for Diagnosis and Treatment.* New York, NY: Guilford Press.

[B7] Bental B. Tirosh E. (2008). The effects of methylphenidate on word decoding accuracy in boys with attention-deficit/hyperactivity disorder. *J. Clin. Psychopharmacol.* 28 89–92. 10.1097/jcp.0b013e3181603f0e 18204348

[B8] Brock S. E. Knapp P. K. (1996). Reading comprehension abilities of children with attention deficit/hyperactivity disorder. *J. Atten. Disord.* 1 173–185. 10.1177/108705479600100305

[B9] Cherkes-Julkowski M. Stolzenberg J. Hatzes N. Madaus J. (1995). Methodological issues in assessing the relationship among ADD, medication effects and reading performance. *J. Learn. Disabil.* 6 21–30.

[B10] De Jong C. G. Van De Voorde S. Roeyers H. Raymaekers R. Allen A. J. Knijff S. (2009). Differential effects of atomoxetine on executive functioning and lexical decision in attention-deficit/hyperactivity disorder and reading disorder. *J. Child Adolesc. Psychopharmacol.* 19 699–707. 10.1089/cap.2009.0029 20035588

[B11] DuPaul G. J. Gormley M. J. Laracy S. D. (2013). Comorbidity of LD and ADHD: implications of DSM-5 for assessment and treatment. *J. Learn. Disabil.* 46 43–51. 10.1177/0022219412464351 23144063

[B12] DuPaul G. J. Stoner G. (2003). *ADHD in the Schools*, 2nd Edn New York, NY: The Guilford Press.

[B13] Evans S. W. Pelham W. E. Smith B. H. Bukstein O. Gnagy E. M. Greiner A. R. (2001). Dose-response effects of methylphenidate on ecologically valid measures of academic performance and classroom behavior in adolescents with ADHD. *Exp. Clin. Psychopharmacol.* 9 163–175. 10.1037/1064-1297.9.2.163 11518092

[B14] Fienup D. M. Reyes-Giordano K. Woloski K. Aghjayan A. Chacko A. (2015). Brief experimental analysis of reading deficits for children with attention-deficit/hyperactivity disorder. *Behav. Modif.* 39 191–214. 10.1177/0145445514550393 25246507

[B15] Forness S. R. Cantwell D. P. Swanson J. M. Hanna G. L. Youpa D. (1991). Differential effects of stimulant medication on reading performance of boys with hyperactivity with and without conduct disorder. *J. Learn. Disabil.* 24 304–310. 10.1177/002221949102400507 2045727

[B16] Forness S. R. Kavale K. A. Sweeney D. P. Crenshaw T. M. (1999). The future of research and practice in behavioral disorders: psychopharmacology and its school implications. *Behav. Disord.* 24 305–318.

[B17] Forness S. R. Swanson J. M. Cantwell D. P. Youpa D. Hanna G. L. (1992). Stimulant medication, and reading performance follow-up on sustained dose in ADHD boys with, and without conduct disorders. *J. Learn. Disabil.* 25 115–123. 10.(1177)/002221949202500205 1583417

[B18] Froehlich T. E. Lanphear B. P. Epstein J. N. Barbaresi W. J. Katusic S. K. Kahn R. S. (2007). Prevalence, recognition, and treatment of attention-deficit/hyperactivity disorder in a national sample of US children. *Arch. Pediatr. Adolesc. Med.* 161 857–864. 10.1001/archpedi.161.9.857 17768285

[B19] Ghelani K. Sidhu R. Jain U. Tannock R. (2004). Reading comprehension and reading related abilities in adolescents with reading disabilities and attention-deficit/hyperactivity disorder. *Dyslexia* 10 364–384. 10.1002/dys.285 15573965

[B20] Grizenko N. Bhat M. Schwartz G. Ter-Stepanian M. Joober R. (2006). Efficacy of methylphenidate in children with attention-deficit hyperactivity disorder and learning disabilities: a randomized crossover trial. *J. Psychiatry Neurosci.* 31 46–51. 16496035 PMC1325066

[B21] Jitendra A. K. DuPaul G. J. Someki F. Tresco K. E. (2008). Enhancing academic achievement for children with attention-deficit hyperactivity disorder: evidence from school-based intervention research. *Dev. Disabil. Res. Rev.* 14 325–330. 10.1002/ddrr.39 19072754

[B22] Kaplan B. Crawford S. Cantell M. Kooistra L. Dewey D. (2006). Comorbidity, co-occurrence, continuum: what’s in a name? *Child Care Health Dev.* 32 723–731. 10.1111/j.1365-2214.2006.00689.x 17018047

[B23] Keulers E. H. Hendriksen J. G. Feron F. J. Wassenberg R. Wuisman-Frerker M. G. Jolles J. (2007). Methylphenidate improves reading performance in children with attention deficit hyperactivity disorder and comorbid dyslexia: an unblinded clinical trial. *Eur. J. Paediatr. Neurol.* 11 21–28. 10.1016/j.ejpn.2006.10.002 17169593

[B24] Lewandowski L. J. Lovett B. J. (2014). “Learning disabilities,” in *Child Psychopathology*, eds Mash E. J. Barkley R. A. (New York, NY: Guilford Press), 625–669.

[B25] Loe I. M. Feldman H. M. (2007). Academic and educational outcomes of children with ADHD. *J. Pediatr. Psychol.* 32 643–654. 10.1093/jpepsy/jsl054 17569716

[B26] Mahone E. M. (2011). *The Effects of ADHD (Beyond Decoding Accuracy) on Reading Fluency and Comprehension, New Horizons for Learning.* Available at: http://education.jhu.edu/PD/newhorizons/Journals/Winter2011/Mahone

[B27] Manset-Williamson G. Nelson J. M. (2005). Balanced, strategic reading instruction for upper-elementary and middle school students with reading disabilities: a comparative study of two approaches. *Learn. Disabil. Q.* 28 59–74. 10.2307/4126973

[B28] McGrath L. M. Pennington B. F. Shanahan M. A. Santerre Lemmon L. E. Barnard H. D. Willcutt E. G. (2011). A multiple deficit model of reading disability and attention-deficit/ hyperactivity disorder: searching for shared cognitive deficits. *J. Child Psychol. Psychiatry* 52 547–557. 10.1111/j.1469-7610.2010.02346.x 21126246 PMC3079018

[B29] Miller A. C. Keenan J. M. Betjemann R. S. Willcutt E. G. Pennington B. F. Olson R. K. (2013). Reading comprehension in children with ADHD: cognitive underpinnings of the centrality deficit. *J. Abnorm. Child Psychol.* 41 473–483. 10.1007/s10802-012-9686-8 23054132 PMC3561476

[B30] Nigg J. T. Barkley R. A. (2014). “Attention-deficit/hyperactivity disorder,” in *Child Psychopathology*, Mash E. J. Barkley R. A. (New York, NY: Guilford Press), 75–144.

[B31] Scanlon D. M. Vellutino F. R. (1996). Prerequisite skills, early instruction, and success in first-grade reading: selected results from a longitudinal study. *Ment. Retard. Dev. Disabil. Res. Rev.* 2 54–63. 10.1002/(SICI)1098-2779(1996)2:1<54::AID-MRDD9>3.0.CO;2-X

[B32] Sesma H. W. Mahone E. M. Levine T. Eason S. H. Cutting L. E. (2009). The contribution of executive skills to reading comprehension. *Child Neuropsychol.* 15 232–246. 10.1080/09297040802220029 18629674 PMC2728040

[B33] Sexton C. C. Gelhorn H. Bell J. Classi P. (2012). The co-occurrence of reading disorder and ADHD: epidemiology, treatment, psychosocial impact, and economic burden. *J. Learn. Disabil.* 45 538–64. 10.1177/0022219411407772 21757683

[B34] Shaywitz B. A. Williams D. W. Fox B. K. Wietecha L. A. (2014). Reading outcomes of children and adolescents with attention-deficit/hyperactivity disorder and dyslexia following atomoxetine treatment. *J. Child Adolesc. Psychopharmacol.* 24 419–425. 10.1089/cap.2013.0148 25299355

[B35] Sheikhi A. R. Martin N. Hay D. Piek J. P. (2013). Phenotype refinement for comorbid attention deficit hyperactivity disorder and reading disability. *Am. J. Med. Genet. B Neuropsychiatr. Genet.* 162 44–54. 10.1002/ajmg.b.32119 23197436

[B36] Snider V. E. Busch T. Arrowood L. (2003). Teacher knowledge of stimulant medication and ADHD. *Remedial Spec. Educ.* 24 46–56. 10.1177/074193250302400105

[B37] Stubenrauch C. Freund J. Alecu de Flers S. Scharke W. Braun M. Jacobs A. M. (2014). Nonword reading and stroop interference: what differentiates attention-deficit/ hyperactivity disorder and reading disability? *J. Clin. Exp. Neuropsychol.* 36 244–260. 10.1080/13803395.2013.878690 24524421

[B38] Sumner C. R. Gathercole S. Greenbaum M. Rubin R. Williams D. Hollandbeck M. (2009). Atomoxetine for the treatment of attention-deficit/hyperactivity disorder (ADHD) in children with ADHD and dyslexia. *Child Adolesc. Psychiatry Ment. Health* 3 1–9. 10.1186/1753-2000-3-40 20003507 PMC2805604

[B39] Tannock R. Martinussen R. Frijters J. (2000). Naming speed performance and stimulant effects indicate effortful, semantic processing deficits in attention-deficit/hyperactivity disorder. *J. Abnorm. Child Psychol.* 28 237–252. 10.1023/A:1005192220001 10885682

[B40] Van Kleeck A. Schuele C. M. (1987). Precursors to literacy: normal development. *Top. Lang. Disord.* 7 13–31. 10.1097/00011363-198703000-00004

[B41] Vaughan B. S. Roberts H. J. Needelman H. (2009). Current medications for the treatment of attention-deficit hyperactivity disorder. *Psychol. Sch.* 46 846–856. 10.1002/pits.20425

[B42] Willcutt E. G. Betjemann R. S. McGrath L. M. Chhabildas N. A. Olson R. K. DeFries J. C. (2010). Etiology and neuropsychology of comorbidity between RD and ADHD: the case for multiple-deficit models. *Cortex* 46 1345–1361. 10.1016/j.cortex.2010.06.009 20828676 PMC2993430

